# Evaluation of the Effect of Different Universal Adhesives on Remineralized Enamel by Shear Bond Strength and Fe-SEM/EDX Analysis

**DOI:** 10.3390/jfb16010023

**Published:** 2025-01-13

**Authors:** Beyza Arslandaş Dinçtürk, Cemile Kedici Alp

**Affiliations:** Restorative Dentistry Department, Faculty of Dentistry, Gazi University, 06510 Ankara, Turkey; cemilealp@gazi.edu.tr

**Keywords:** bond strength, EDX analysis, universal adhesives, tooth remineralization

## Abstract

The aim of this study is to evaluate the shear bond strength of different universal adhesives applied to intact, demineralized, and remineralized enamel surfaces with total-etch and self-etch modes and to examine the effect of universal adhesives on the Ca/P mineral atomic and mass ratios of these enamel with FE-SEM/EDX (Field Emission Scanning Electron Microscopy with Energy Dispersive X-Ray Spectroscopy) analysis. For this study, 264 bovine incisors were used. Samples in the demineralized and remineralized groups were kept in demineralization solution at 37 °C for 96 h to make an artificial initial carious lesion. After demineralization, half of the demineralized samples were remineralized with MI Paste Plus. For shear bond strength (n = 144) and FE-SEM/EDX analysis (n = 120), G-Premio Bond and Clearfil S^3^ Bond Universal were applied on enamel surfaces with total-etch and self-etch modes, and bond strength samples were restored with resin composite. All samples were tested. The results were evaluated statistically by a three-way ANOVA test. The shear bond strength of the remineralized enamel showed high bond strength values comparable to intact enamel for universal adhesive systems. The Ca/P mineral atomic and mass ratios in remineralized enamel showed higher values than demineralized enamel, similar to intact enamel for universal adhesive systems. Initial carious lesion surfaces are unsuitable enamel surfaces for restoration. The remineralization of this surface layer before adhesive procedures may positively affect bond strength.

## 1. Introduction

Dental caries remains a significant global public health concern. The process of caries starts with molecular changes in the tooth enamel’s hydroxyapatite crystals and is accompanied by demineralization, which occurs with the loss of calcium and phosphate ions from the tooth structure as a result of the imbalance between protective and pathological factors [1]. Rather than a one-way demineralization process, caries arises from the accumulation of numerous demineralization and remineralization attacks [2]. Clinically, the first noticeable symptom is white spot lesions (WSLs) on the enamel, which may progress to subsurface enamel lesions and dentin cavitation formation.

Demineralization begins below the surface layer of enamel and causes an increase in porosity. The enamel surface layer usually remains intact during demineralization [3]. The surface of the initial enamel caries lesion is solid, intact, and opaque–white in appearance, and it has not yet reached the enamel–dentin junction [4]. The reversal of demineralization can be achieved when sufficient calcium and phosphate ions are present around the tooth. Thus, dissolved hydroxyapatite crystals are remineralized and leading to lesion repair.

Bonding studies are an important parameter in the evaluation of restorative materials. Just as crucial as the restorative materials are the bonded surfaces. There are structural differences between intact and demineralized enamel, which affects the bonding of restorative materials to enamel and compromises clinical success. Studies in the literature [5,6] indicate that bonding to demineralized enamel is a clinical challenge. For this reason, demineralized lesions need to be treated. Remineralization as a non-invasive treatment for initial carious lesions represents a significant advancement in the clinical management of dental caries. Demineralized lesions can be remineralized with a variety of agents [5,6,7,8]. It is claimed that products containing fluoride, phosphate, and calcium increase remineralization more than products containing only fluoride [9]. In addition to fluoride, the ingredients which provide a rise in the resistance to acid dissolution, calcium, casein, and phosphate ions, also provide remineralization. The casein phosphopeptide (CPP) component of the casein phosphopeptide–amorphous calcium fluoro phosphate (CPP-ACP(F)) complex readily binds to tooth enamel, biofilm, and soft tissues when CPP-ACP(F) is applied. This allows calcium and phosphate ions to be delivered to the necessary locations. Free calcium and phosphate ions leave CPP, reach the enamel prisms, and re-form apatite crystals, providing remineralization of deeper areas of the lesion [10]. It has been reported that tooth enamel applied with CPP-ACP(F) is superior to products containing only fluoride in terms of acid resistance and caries prevention [11]. However, it is thought that this acid-resistant enamel surface may affect adhesion, and the residual CPP-ACP(F) remaining on the enamel surface cannot move away and accumulates on the surface, preventing the bonding between the adhesive system and the enamel [12]. According to certain studies, the bond strength between restorative materials and demineralized enamel is less than that of intact and remineralized enamel [8,13].

Universal adhesives are frequently preferred in studies evaluating bond strength to enamel and dentin due to their advantages such as ease of use, low technical sensitivity, faster application, and applicability with both self-etch and total-etch modes. Universal adhesives are used in dentistry based on how acidic they are; they are classified into four groups: strong (pH < 1), medium (1 < pH < 2), light (pH ≈ 2), and ultra-light (pH > 2.5) [14,15]. The adhesive content used is as important as the bonded surface on which the adhesive is applied. All adhesive systems exhibit suitable bond strength independent of application methods [16,17,18,19]. The performance of self-etch systems is debatable, but universal adhesives have a stronger bond strength when used with the total-etch mode on enamel because they involve a separate etching step. Different studies use different parameters, like type of acid, acid concentration, application time, application method, and bond strength test. The composition of the adhesive system, including difference in acetone, alcohol, ethanol, and water content, can also significantly impact adhesive bond strength. According to the literature, a few studies have been conducted to assess the bond strength of universal adhesives with varying acidities to intact, demineralized, and remineralized enamel using the modes of self-etch and total-etch [5]. In addition, there was no information about the effect of universal adhesives with different acidities on the change in surface Ca/P atomic and mass ratios after application to enamel surfaces with the modes of self-etch and total-etch. Thus, the purpose of this study was to assess universal adhesive systems’ shear bond strength with different acidities applied to intact, demineralized, and remineralized enamel surfaces with self-etch and total-etch modes and to assess the ratios of Ca/P with FE-SEM/EDX analysis. This study’s first null hypothesis is as follows: different application modes (total-etch and self-etch) of Clearfil S^3^ Bond Universal and G-Premio Bond Universal adhesive systems on different enamel (intact, demineralized, and remineralized) tissues will not influence the shear bond strength of these adhesives. The second null hypotheses of this study is as follows: the values of the Ca/P atomic and mass ratios will not differ in the EDX analysis of Clearfil S^3^ Bond Universal and G-Premio Bond Universal adhesive systems applied to intact, demineralized, and remineralized enamel surfaces with total-etch and self-etch modes.

## 2. Materials and Methods

The material contents and usage methods utilized in this study are shown in Table 1.

### 2.1. Selection and Preparation of Teeth

In total, 264 sound bovine incisors were used for this study. The teeth were stored in 0.01% thymol solution following extraction (144 for shear bond strength test and 120 for EDX analysis). After extraction, teeth were washed thoroughly, periodontal tissues were removed with hand tools, and teeth were cleaned with a low-speed micromotor. The crown portion of the teeth was separated by cutting them at the cemento-enamel junction level. Enamel samples of 5 × 5 mm^2^ were obtained from the middle of the teeth’s buccal surfaces with a diamond bur.

**Figure 1:**
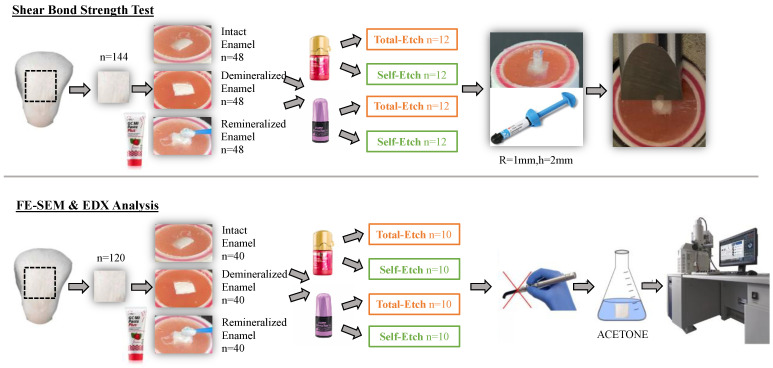
Flow chart.

Enamel samples were separated into 3 main groups: the first group consists of the intact enamel group without any pretreatment (control group), the second group consists of the demineralized enamel group, and the third group consists of the enamel group to which a remineralizing agent containing CPP-ACP(F) was applied to the previously demineralized enamel. Each main group’s enamel samples were divided into four subgroups based on the various adhesive systems and application modes used (Figure 1).

### 2.2. Shear Bond Strength Test

#### 2.2.1. Preparation of Samples

Enamel sample was placed in PVC tubes and filled with acrylic resin (IMICRYL, Konya, Turkey), leaving the teeth’s buccal surfaces uncovered. Enamel surfaces were standardized using silicon carbide abrasive sandpaper (600-grit) for 30 s to create a homogeneous smear layer and then rinsed with distilled water for 30 s.

#### 2.2.2. Procedure of Demineralization and Remineralization

The initial values of the enamel samples were measured and recorded using DIAGNOdent Pen (KaVo Dental GmbH, Biberach/Riß, Germany). The first group was reserved for use in intact enamel groups (control group) without any pretreatment (n = 48). Samples in the demineralized and remineralized groups (n = 96) were demineralized to create an artificial initial caries lesion. Samples were kept in 40 mL of demineralization solution consisting of 2.2 mM calcium chloride dihydrate (CaCl_2_), 2.2 mM sodium hydrogen phosphate (NaH_2_PO_4_), and 50 mM acetic acid with a pH of 4.2 used in this study and 1 M KOH for pH adjustment [5].

Samples were kept in demineralization solution and the demineralization procedure was continued for 96 h at 37 °C, and the solution was prepared fresh and replaced every day. After 96 h, the samples were removed and washed with distilled water for 30 s, followed by drying for 10 s. A total of 96 samples were confirmed with demineralization, and 48 samples were separated for remineralization in the third group. For the samples in this group, the tooth surface was treated with the MI Paste Plus agent in accordance with Table 1 and left for 4 min [10] (Figure 1) and then washed away with deionized water for 30 s [20]. Deionized water was drawn into the syringe as 30 mL for each sample and applied perpendicular to the surface with finger pressure so that standardization was ensured. After that, the samples were stored in artificial saliva for one week [10]. The confirmation of demineralization and remineralization was achieved using the DIAGNOdent Pen. The device was calibrated before each measurement. Then, the samples were randomly distributed to 4 subgroups (G-Premio Bond universal with total-etch mode, G-Premio Bond universal with self-etch mode, Clearfil S^3^ Bond Universal with total-etch mode, and Clearfil S^3^ Bond Universal with self-etch mode) (n = 12).

#### 2.2.3. Adhesive Procedures

After different enamel surfaces were prepared, adhesive procedures were applied to the enamel as follows:

- In the total-etch mode, 37% phosphoric acid gel was applied for 30 s, washed, and the enamel surface was dried. Universal adhesives were applied with the total-etch mode as previously described in Table 1 and polymerized for 10 s with D-Light Pro LED light sources (1400 mW/cm^2^). Resin composite (Filtek™ Ultimate Universal) was placed at a height of 2 mm using transparent polyethylene tubes with an inner diameter of 2 mm and polymerized with a D-Light Pro LED device for 20 s.
- In the self-etch mode, universal adhesives were applied as previously described in Table 1 and polymerized for 10 s with a D-Light Pro LED light device. Resin composite (Filtek™ Ultimate) was placed at a height of 2 mm using transparent polyethylene tubes with an inner diameter of 2 mm and polymerized with a D-Light Pro LED device for 20 s.

The PVC tubes were then carefully taken out using a No. 11 scalpel. Samples were kept in distilled water for 24 h at 37 °C for 1 day and then they were subjected to a shear bond strength test.

#### 2.2.4. Shear Bond Strength Test

Shear bond strength samples were tested using a universal testing machine (Shimadzu AG-IS Autograph, Shimadzu Scientific Instruments, Kyoto, Japan). The crosshead speed was set at 1 mm/min until the sample failed, separating the composite cylinder from the enamel surface. Shear bond strength values (MPa) were calculated by dividing the load at fracture by the surface area (mm^2^).

#### 2.2.5. Failure Types Analysis

Following the shear bond strength testing, samples were examined at 100× magnification in a stereomicroscope (SZH-131, Olympus Ltd., Tokyo, Japan) to determine failure types. Failure types were determined as four categories [21] and named according to ISO 10365:2022 [22]:

- Type AF: Failure of the adhesive type (enamel/adhesive interface);
- Type CSF: Failure of the cohesive type (within enamel);
- Type CF: Failure of the cohesive type (within composite);
- Type ACFP: Failure of mixed type (partial cohesive and partial adhesive failure).

### 2.3. Enamel Etching Pattern and EDX Analysis

#### 2.3.1. Preparation of Samples

Samples for the enamel etching pattern and EDX analysis were prepared in the same way as the samples for the shear bond strength test.

#### 2.3.2. Procedure of Demineralization–Remineralization

The initial values of the prepared enamel samples were measured and recorded using the DIAGNOdent Pen. As previously described, intact (n = 40), demineralized (n = 40), and remineralized (n = 40) samples were prepared. The surfaces after demineralization and remineralization were measured using the DIAGNOdent Pen (Table 2). Samples were randomly distributed into subgroups. In total, 40 intact samples were divided into 4 subgroups (as previously mentioned) (n = 10), and FE-SEM images were recorded to evaluate the effect of universal adhesives on different enamel surfaces by enamel etching pattern, and the Ca/P atomic and mass ratio was evaluated by EDX analysis.

#### 2.3.3. Enamel Etching Pattern

The enamel etching pattern (n = 10) was evaluated on the enamel surface using Field Emission Scanning Electron Microscopy (FE-SEM; Hitachi Su5000 Hitachi Ltd., Chiyoda-ku, Japan). The accelerator voltage used was 10 kV, and the beam current was 131 µA. A secondary electron detector was used as the imaging detector. The vacuum level of the chamber was <1 × 10^−3^ Pa. Spot intensity 30 was used to observe the surface. The WD (working distance) was changed between 10 mm and 15 mm. For this purpose, universal adhesive systems were applied to demineralized, remineralized, and intact enamel surfaces, as described in Table 1. Phosphoric acid gel, applied only in total-etch mode, was applied to the enamel for 30 s, washed for 10 s, and air-dried according to the manufacturer’s instructions. Universal adhesive systems were not polymerized; to dissolve the resin material on the enamel surfaces, it was quickly placed in 100% acetone [24] after application and stored in acetone for 24 h. Then, to remove the resin from the samples, it was washed and rinsed with deionized water for 5 min, with 96% alcohol for 5 min, and again with deionized water for 5 min. All samples were dried in a desiccator for 12 h and sputter-coated with gold in a vacuum coating device (Leica Ace 200, Leica Microsystems, Wetzlar, Germany).

#### 2.3.4. Energy Dispersive X-Ray Spectroscopy Analysis (EDX)

Regarding the semiquantitative chemical microanalysis of Ca/P atomic and mass ratios using EDX, demineralized, remineralized, and intact enamel samples (n = 10) were evaluated under a field emission scanning electron microscope (Figure 1). Xmax-80 of Oxford instruments (Abingdon, UK) was used as an EDX detector. The accelerating voltage and beam current are the same as those used with the SEM parameters. And, to more accurately count the data, the spot intensity is adjusted to 50. Three points per sample were randomly selected, and Ca/P atomic and mass ratios were examined in three points using EDX analysis. Ca/P ratios were recorded, and the mean values were calculated [25]. Microphotographs of representative surface areas were taken at 2500× magnification.

#### 2.3.5. Statistical Analysis

G*Power 3.0.10 (Franz Faul, Universität Kiel, Kiel, Germany) software was used to calculate the sample size; the type 1 error (α) was 0.05, and the effect size was 0.4. With an analysis power of 0.90, 12 teeth would be provided for each group. The Shapiro–Wilk test was used to study the data, and the data were examined to determine whether the parameters were suitable for a normal distribution. The results showed that the parameters did exhibit a normal distribution. For homoscedasticity, Levene’s test was used, and it was determined that the data were homogeneous (*p* > 0.05). In order to assess the impact of enamel surface, application method, and universal adhesive on bond strength and Ca/P atomic and mass ratios, a three-way ANOVA and Tukey’s post hoc HSD test were employed. The significance level was set at *p* < 0.05.

## 3. Results

### 3.1. DIAGNOdent Pen Values

The DIAGNOdent Pen group mean values are shown in Table 2.

In this study, the baseline, post-demineralization, and post-remineralization fluorescence values of intact enamel were measured with the DIAGNOdent Pen (Table 2). When the remineralization capacity of MI Paste Plus (used to remineralize or demineralize the surfaces) was evaluated with the DIAGNOdent Pen, it was observed that MI Paste Plus significantly increased remineralization and approached the initial intact enamel reference values. Samples that were not among the reference values were not included in this study.

### 3.2. Shear Bond Strength

Averages and standard deviations of the data obtained as a result of the shear bond strength test are shown in Table 3.

**Figure 2:**
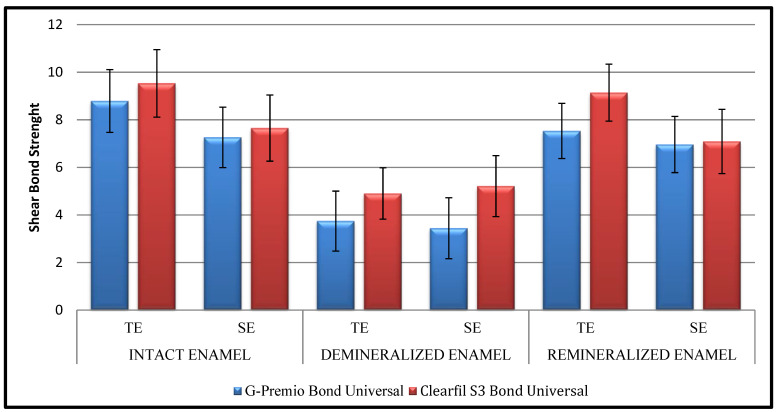
Bond strength values (TE: total-etch; SE: self-etch).

In both universal adhesive systems, the highest bond strength in all modes was obtained in intact enamel, then in remineralized enamel, and the lowest was obtained in demineralized enamel (Table 3). In the intact enamel groups, the bond strength of G-Premio Bond Universal (*p*: 0.009; *p* < 0.05) and Clearfil S^3^ Bond Universal (*p*: 0.003; *p* < 0.05) with total-etch mode was higher than the self-etch mode (*p*: 0.009; *p* < 0.05). In the remineralized enamel groups, the bond strength of Clearfil S^3^ Bond Universal total-etch mode was higher than self-etch mode (*p*: 0.001; *p* < 0.05); there was no statistically significant difference between the bond strengths of the G-Premio Bond groups (*p*: 0.242; *p* > 0.05). In the demineralized enamel groups, there was no statistically significant difference between the bond strengths of the G-Premio Bond Universal groups (*p*: 0.565; *p* > 0.05) and between the Clearfil S^3^ Bond Universal groups (*p*: 0.526; *p* > 0.05) (Figure 2). 

The stereomicroscopic evaluation of the failure types of the Clearfil S^3^ Bond Universal and G-Premio Bond Universal fracture surfaces showed that the most common failure type was the adhesive type (46.5%), followed by the mixed type (36.2%) and cohesive failure type in enamel (11.8%), and the least common was the cohesive type in composite (5.5%). A higher rate of adhesive type failure was observed in the groups with high bond strength values. The distribution of failure types between the groups is shown in Table 4. In the demineralized and remineralized groups, cohesive failure types were observed, unlike in the intact enamel groups.

### 3.3. SEM/EDX

#### 3.3.1. Enamel Etching Pattern (FE-SEM)

**Figure 3:**
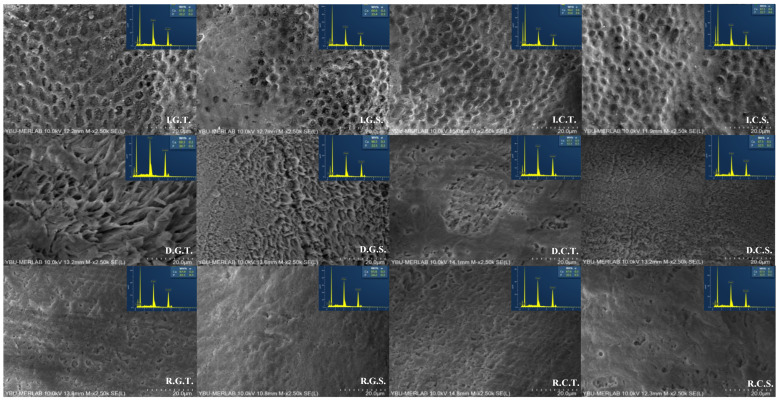
FE-SEM images of universal adhesives when applied on different enamel surfaces with different modes (I: intact enamel, D: demineralized enamel, R: remineralized enamel, G: G-Premio Bond Universal, C: Clearfil S^3^ Bond Universal, T: total-etch, S: self-etch) (magnification: 2.5 k, the bar size: 20 μm).

Enamel surfaces, from the FE-SEM analysis at 2500× magnification, are shown in Figure 3. In the FE-SEM image analysis, in self-etch mode on the intact enamel surface for both adhesive systems, smaller surface morphology changes, slight roughening signs, and shallow pits were observed in some areas (I.G.S. and I.C.S.) compared to when applied in total-etch mode. With the total-etch mode in intact enamel, a deeper demineralization was observed in both adhesive systems (I.G.T. and I.C.T.). Adhesive systems applied with the total-etch mode, regardless of the enamel surface, produced the deepest and most significant demineralization compared to the self-etch mode. When demineralized enamel surfaces are evaluated, more surface degradation is observed in both adhesive systems with the total-etch mode (D.G.T. and D.C.T.) than with the self-etch mode. In the groups where MI Paste Plus was applied, the demineralized enamel surface was covered, and when applied with the total-etch mode (R.G.T. and R.C.T.), enamel porosities were more evident due to the effect of etching. The morphology changes observed in the G-Premio Bond Universal groups are more than those in the Clearfil S^3^ Bond Universal groups. It was observed that G-Premio Bond Universal creates more surface demineralization when applied with the self-etch and total-etch modes in intact enamel groups.

#### 3.3.2. Energy Dispersive X-Ray Spectroscopy Analysis (Ca/P Atomic and Mass Ratios)

Averages and standard deviations of the Ca/P mineral atomic and mass ratios obtained as a result of the EDX analysis are shown in Table 5 and Table 6.

In both universal adhesive systems, the highest Ca/P atomic and mass ratio in self-etch and total-etch modes was obtained in intact enamel, then in remineralized enamel, and the lowest was obtained in demineralized enamel (Table 5 and Table 6). When G-Premio Bond Universal (*p*: 0.001) and Clearfil S^3^ Bond Universal (*p*: 0.001) were applied to the intact and demineralized enamel surface with total-etch and self-etch modes; the average Ca/P atomic and mass ratios of the self-etch mode were statistically significantly higher than the total-etch mode (*p* < 0.05). When G-Premio Bond Universal (*p*: 0.062) and Clearfil S^3^ Bond Universal (*p*: 0.503) were applied to the remineralized enamel surface with self-etch and total-etch modes, there was no statistically significant difference in terms of the average Ca/P atomic and mass ratios between the self-etch and total-etch modes (*p* > 0.05).

## 4. Discussion

In the literature, it can be seen that extracted bovine incisors [8,13,21] and human teeth [5,6,26] were used in in vitro studies. Bovine and human tooth enamel tissue have a similar evolutionary origin. Considering the similarity of bovine teeth to the microstructure of human tooth enamel, bovine teeth can be used as an alternative to human teeth in research [27,28,29,30,31]. Studies have shown that shear bond strength values show equal or close values between bovine and human enamel [29,30,31]. Bovine teeth are easier and less time-consuming to obtain than human teeth and are easy to reproduce. In addition, larger surfaces can be obtained from bovine teeth. Due to their similar physicochemical characteristics and chemical compositions, bovine and human enamels share many characteristics [32]. The carbonate contents, physical characteristics, polishing ability, luminescence, and refractive indices of human and bovine enamel do not differ significantly. According to Davidson et al. [33], the calcium distribution in bovine enamel is more uniform than in human enamel, and the calcium contents of the two types of enamel were 37.9% (bovine) and 36.8% (human) by weight, respectively. Feagin et al. [34] reported that human and bovine enamel share similar demineralization and remineralization properties. Since bovine tooth enamel is used in remineralization studies as well as bond strength studies [35,36], bovine incisor tooth enamel was used in this study. In remineralization studies, it is important to be able to create a demineralized initial carious lesion under in vitro conditions. Various demineralization solutions are used for this purpose [5,8,13,37]. In this study, demineralization solution consisting of 2.2 mM calcium chloride dihydrate (CaCl_2_), 2.2 mM sodium hydrogen phosphate (NaH_2_PO_4_), and 50 mM acetic acid with a pH of 4.2 was preferred [37], and after immersion in this solution, demineralization was confirmed with the DIAGNOdent Pen device. The DIAGNOdent Pen is used as a reliable parameter in many studies, including caries detection and remineralization studies [38,39,40,41,42,43]. The reproducibility of the device in vitro and in vivo is important to confirm that consistent measurements can be taken, which proves that it can be used for demineralization–remineralization monitoring [25,43]. It works with a mechanism based on the increase in the fluorescence of the changes that occur on the surface with caries. This fluorescence is displayed on the DIAGNOdent Pen with numbers ranging from 0 to 99. In this study, lesions were confirmed with the DIAGNOdent Pen, and specimens with enamel demineralization not within the reference range (enamel caries: 7–17) measured with the DIAGNOdent Pen after 96 h in demineralization solution were excluded (Table 2) [25].

Clearfil S^3^ Bond Universal and G-Premio Bond Universal adhesive systems with varying acidities were tested for shear bond strength on different enamel tissues (intact, demineralized, and remineralized) using different application methods (total-etch and self-etch). The results showed that Clearfil S^3^ Bond Universal and G-Premio Bond Universal adhesive systems applied with the total-etch mode provided higher bond strength to intact and remineralized enamel than those applied with the self-etch mode. In addition, the values of shear bond strength for G-Premio Bond Universal (pH 1.8) and Clearfil S^3^ Bond Universal (pH 2.7) adhesive systems applied to intact, demineralized, and remineralized enamel surfaces varied, which led us to reject the first null hypothesis.

In this study, the lowest shear bond strength values were recorded when Clearfil S^3^ Bond Universal and G-Premio Bond Universal adhesives bonded to demineralized enamel. This supports the findings of earlier research [5,8]. This could be explained by the low quality of the demineralized enamel surface, which obstructs the appropriate micromechanical interlock. It is also thought to be caused by the removal of elements such as calcium from the surface [44]. The mineral loss on the enamel due to the higher P and Ca loss from the surface compared to intact and remineralized enamel can be used to explain the insufficient bond strength observed on the demineralized enamel surface. Furthermore, appropriate micromechanical locking may be limited by the demineralized enamel’s current poor-quality enamel surface. When using self-etch and total-etch modes on demineralized enamel surfaces, Clearfil S^3^ Bond Universal shows greater bond strength than G-Premio Bond Universal (*p* < 0.05). HEMA-free adhesives that are less hydrophilic have been developed in an effort to lessen the hydrolytic degradation of adhesives. But HEMA’s solvation effect is also eliminated. G-Premio Bond Universal does not contain HEMA monomers, in contrast to Clearfil S^3^ Bond Universal, which contains HEMA monomers. When other solvents, such as acetone or ethanol, evaporate, water tends to separate the adhesive components, making these adhesives prone to phase separation [45,46]. The resulting water blisters may lower the immediate bond strength. The studies examining the durability of bond strength with HEMA-free adhesives are limited and the results are conflicting, but generally, loss of bond strength seems to occur [47,48,49].

There was no statistically significant difference between Clearfil S^3^ Bond Universal with a pH of 2.7 and G-Premio Bond Universal with a pH of 1.8 in self-etch and total-etch modes in intact enamel. Although statistically insignificant, the bond strength of Clearfil S^3^ Bond Universal was higher. This result is possibly attributed to the high water content in G-Premio Bond Universal and the different chemical contents of the adhesives. Strong self-etch adhesive systems contain a more acidic resin monomer and a higher amount of water [15]. G-Premio Bond Universal, one of the universal adhesives tested, has a pH of 1.8 and a water content of about 24%. It has been reported that G-Premio Bond Universal contains a high amount of water to enhance the demineralization of tooth surfaces, while a low water concentration leads to a hydrophobic adhesive interface resistant to hydrolytic degradation [15]. Excess water content in adhesive systems is thought to adversely affect polymerization, leading to inadequate bonding [50]. G-Premio Bond Universal does not contain hydrophilic HEMA monomers, and this may be the reason for its lower bond strength, even though it was statistically insignificant when compared to Clearfil S^3^ Bond Universal when applied to intact enamel using self-etch and total-etch modes. The acidic monomer in Clearfil S^3^ Bond Universal may have led to a deeper demineralization of the tooth surface because of the HEMA.

In previous studies [39,51] using different demineralization and remineralization protocols, similar findings were obtained for bond strength on enamel remineralized using CPP-ACP and CPP-ACP(F). The bond strength of Clearfil S^3^ Bond Universal and G-Premio Bond Universal to remineralized enamel is close to intact enamel, but there is a statistically significant difference only when G-Premio Bond Universal is applied with the total-etch mode (pR-S:0.045; *p* < 0.05). It can be concluded that the use of CPP-ACP(F) provides bond strength in demineralized enamel to a level comparable to intact enamel. Applying CPP-ACP(F) to the enamel’s surface allows it to react with hydrogen ions to create calcium hydrogen phosphate, which releases calcium and phosphate ions and protects the enamel from acid-induced dissolution [10]. It has been reported that enamel treated with CPP-ACP(F) is more acid-resistant and superior in reducing the risk of caries compared to products containing only fluoride [11]. In this study, CPP-ACP(F)-applied enamel groups showed statistically significantly higher bond strength values than demineralized enamel groups. CPP-ACP(F) fills in the gaps left by demineralization on the tooth surface, preventing demineralization, promoting remineralization, and creating Ca accumulation on the surface of the enamel, whereas hardness declines and mineral is lost as a result of demineralization. 10-MDP monomer in universal adhesives may have formed an ionic bond with the Ca on the surface. The high bond strength values in remineralized enamel can be attributed to the ionic bond between 10-MDP-Ca due to both the Ca from enamel and the Ca from CPP-ACP(F). This acid-resistant enamel surface can affect adhesion, and the residual CPP-ACP(F) complex cannot move away from the enamel surface and come together on the surface to prevent the adhesion between the adhesive system and enamel. It is believed that the remineralized enamel surface does not always have enamel prisms and occasionally consists of a dense mixture of calcium phosphate and fluoride [5,12]. In this study, the bond strength of universal adhesives on enamel surfaces where CPP-ACP(F) was applied approached that of intact enamel. This may be due to the inability of both adhesive systems to effectively abrade the acid-resistant enamel surface or the presence of areas in the remineralization layer that are not sufficiently remineralized. However, the higher bond strength obtained with the total-etch mode compared to the self-etch mode in remineralized enamel groups is possibly due to the fact that the acid etching step applied in total-etch systems acts on this acid-resistant layer and erodes the enamel surface. When the total-etch mode is applied to the enamel surface remineralized with MI Paste Plus, the bond strength of Clearfil S^3^ Bond Universal is statistically significantly higher than G-Premio Bond Universal (*p* < 0.05). This increased bond strength can be attributed to the high water content in G-Premio Bond and the different chemical contents of the adhesives, similar to intact enamel.

The stereomicroscopic evaluation of the failure type analysis of Clearfil S^3^ Bond Universal and G-Premio Bond Universal showed that the most common failure type was the adhesive type (46%), followed by the mixed type (42%), and the least common was the cohesive type (12%). A higher rate of adhesive failure is observed in groups with high bond strength values. In this study, for both universal adhesive systems, the most common mixed type failure was observed in demineralized enamel groups. Unlike the intact enamel group, cohesive type failure was observed in demineralized–remineralized enamel samples. The reason for the cohesive failure observed in the demineralized groups can be attributed to the ruptures observed due to the loss of hardness on the demineralized enamel surface and the fragility of the demineralized surface, which is more likely to break under stress. These findings are also compatible with the lowest bond strength values seen in demineralized enamel groups. The most common adhesive type failure was observed in intact enamel groups, where the high rate of adhesive type failure can be attributed to the fact that the breakage originates from the layer at the adhesive–enamel interface due to the high bond strength. For both universal adhesive systems, adhesive and mixed type failures were observed at equal rates in the groups remineralized with MI Paste Plus. The reason for the cohesive failure in groups remineralized with MI Paste Plus may be due to the deterioration that may occur on the remineralized surface due to the acidity of the adhesive.

There are studies in the literature that use the acid etch pattern method to study the impact of adhesives on enamel after application [26,52], but none assess the adhesives’ impact on remineralized enamel. For this reason, in this study, the effect of universal adhesives of different acidities on intact, demineralized, and remineralized enamel surfaces was evaluated with an enamel etching pattern. The purpose of the enamel etching pattern is to view and examine under FE-SEM the pattern created by the acid in the adhesive system applied to the enamel surface after the resin is removed. FE-SEM analysis is widely used in remineralization studies and aids in the observation of surface ultra-morphological changes in dental tissues. In the FE-SEM image analysis, when self-etch mode was applied in each enamel types, less surface morphology change was observed on the enamel morphology compared to the total-etch groups; slight roughening signs and shallow pits were observed in some areas. In self-etch mode, due to the shallow penetration of monomers between crystals and the formation of insufficient resin tags between the prisms, a reduced potential for micromechanical locking is observed, and a lower shear bond strength to the enamel may occur [26]. In the total-etch etching groups of this study, phosphoric acid increased demineralization on the surface, more hydroxyapatite dissolution from enamel prisms and deeper, irregular fossae and grooves were observed, and total-etch can completely remove the smear layer from enamel. When demineralized enamel was evaluated, it was seen that the surface was more deteriorated with total-etch mode. This deterioration can be explained by the increase in rough areas due to mineral and protein loss from the enamel due to demineralization. Porosity appears to be reduced in enamel groups where MI Paste Plus was applied compared to demineralized enamel groups. This may indicate that CPP-ACP(F) blocks and covers the enamel surface in groups where MI Paste Plus was applied. In the total-etch mode, enamel porosities were more evident due to the effect of etching, and in the self-etch mode, porosities were also observed, albeit slightly. In this study, CPP-ACP(F) accumulations were observed on the surface by FE-SEM analysis. Morphology changes observed in the G-Premio Bond Universal groups are more than those that occurred in the Clearfil S^3^ Bond Universal groups. G-Premio Bond Universal shows a deeper and more pronounced etching pattern when applied with self-etch and total-etch modes on intact enamel groups. This may be attributed to the pH of the adhesive being more acidic.

Confirmation of the remineralization of the initial carious lesions and quantitative assessment of the alteration in surface Ca/P atomic and mass ratios can be achieved with EDX analysis. The objective was to remove the adhesive from the surface in this manner. The samples were immediately immersed in pure acetone, the samples were not polymerized, and then they were left there for a day and adhesive was removed. According to the results of this study, the Ca/P mineral atomic and mass ratios of Clearfil S^3^ Bond Universal and G-Premio Bond Universal adhesive systems applied to intact, demineralized, and remineralized enamel surfaces with different application modes (total-etch and self-etch) differ statistically. For this reason, the second null hypothesis was rejected.

Regardless of adhesive application, even when the adhesive layer was removed on the enamel surfaces remineralized with MI Paste Plus, the Ca/P mineral atomic and mass ratios were observed to be higher than in demineralized enamel, approaching the intact enamel surface. In remineralized groups, when G-Premio Bond Universal and Clearfil S^3^ Bond Universal were applied, there was no difference between self-etch mode compared to total-etch mode. In demineralized and intact enamel groups, when applied in self-etch mode, both adhesives showed high Ca/P atomic and mass ratios. These findings support that 37% phosphoric acid reduces the surface Ca/P atomic and mass ratio when the total-etch mode is applied to intact enamel. Acidic monomers have less effect on the Ca/P atomic and mass ratios when applied with the self-etch mode. It may have dissolved less Ca and P from the surface than phosphoric acid. G-Premio Bond Universal showed lower Ca/P atomic and mass ratios compared to Clearfil S^3^ Bond Universal in the intact, demineralized, and remineralized groups. This finding may be due to the fact that Clearfil S^3^ Bond Universal removes less Ca and P from the enamel because of the effect of an ultra-mild acidic monomer in the adhesive.

Kamath et al. [53] compared the potential of CPP-ACP(F) and different remineralization agents on white spot lesions and evaluated the enamel etching pattern by SEM evaluation. Cardenas et al. [26] examined the enamel etch pattern of universal adhesives on intact and fluorotic enamel surfaces and kept samples in pure acetone. Similarly to the studies of Cardenas et al. [26], after applying adhesive systems with different acidities, the resin was removed and FE-SEM/EDX analysis was performed, and similar to the studies of Kamath et al. [53], the surfaces were remineralized. When these remineralized surfaces were examined under EDX, after the application of G-Premio Bond Universal and Clearfil S^3^ Bond Universal adhesive systems, Ca/P mineral atomic and mass ratios were observed to be higher than demineralized enamel and close to intact enamel. Higher Ca/P mineral atomic and mass ratio values were obtained in Clearfil S^3^ Bond Universal groups compared to G-Premio Bond Universal groups.

There are limitations to this study. Despite the similarities between bovine and human teeth, the bonding values may be different due to differences in the prism structure in enamel. This might not be directly generalizable to clinical practice. The environments that caused caries and promoted remineralization were entirely chemical, and the possible blocking effects of an acidic bacterial environment on the observed mechanism were ignored. This study attempted to demineralize enamel under in vitro conditions to create initial carious lesions, which may not fully represent reality. In vitro remineralization may differ from the dynamic and biological remineralization that usually occurs in vivo in the oral environment. Therefore, it may not directly mimic clinical conditions. In this study, MI Paste Plus was applied for 4 min, which may not be sufficient considering the in vivo remineralization dynamics that require longer durations, and longer-duration studies may be needed. Mineral alterations have only been assessed on one surface by FE-SEM analysis. Changes should be detected in detail, including the interface, and data should be obtained from different depths and can be confirmed using different analysis tools. Furthermore, the classification of failure types (adhesive, cohesive, or mixed) was performed with a stereomicroscope at 100× magnification, which may be insufficient to accurately distinguish between truly cohesive failures and more complex mixed failure components. The sample size in this study was justified using G*Power software, but the use of a three-way ANOVA may not be robust enough to detect significant differences, increasing the risk of type II error. Ca/P ratios were analyzed in this study, but other chemical parameters such as the presence of fluoride, enamel hardness, or crystallographic changes in enamel prisms were not evaluated, which may be recommended for future studies. Future studies combining in vitro and in vivo approaches with different analysis tools are needed.

## 5. Conclusions

1. The demineralized layer of enamel, which is present in early caries lesions and white spot lesions resulting from orthodontic treatment, decreases bond strength and is an inappropriate surface for enamel restoration. To improve bond strength in this surface layer, it is recommended to remineralize the surface before adhesive procedures.
2. Bonding to remineralized enamel showed bond strength values close to intact enamel for both universal adhesives. Remineralization agent applied to the surfaces increases the bond strength.
3. Factors other than acidity play a role in the bonding of universal adhesives to remineralized enamel.
4. Ca/P mineral atomic and mass ratios were highest in intact enamel and then in remineralized enamel. Ca/P mineral atomic and mass ratios close to intact enamel were obtained for both universal adhesives. It can be said that the MI Paste Plus agent used for remineralization confirms the remineralization capacity.

## Figures and Tables

**Table 1:** Contents and usage methods of the materials used in this study.

Materials	Contents	Usage
**MI Paste Plus—GC, RECALDENT, Alsip, USA**	CCP-ACP, 900 ppm F-, sodium carboxymethyl cellulose and TiO_2_ with pH: 7	The teeth are covered with a thin and uniform layer, and this is left on the enamel surface for a minimum of 3 min.
**G-Premio Bond Universal—GC Corporation, Tokyo, Japan**	10-MDP, 4-MET, MEPS, methacrylate monomer, acetone, water, silica, initiators **(pH: 1.8)**	**Self-etch** Applied using a micro brush. After the application, adhesive left on the surface for 10 s. Dried with oil-free air under maximum air pressure for 5 s. Cured with light sources at 1200 mW/cm^2^ for 10 s. **Total-etch** Phosphoric acid gel is applied for 30 s. Wash for 10 s, rinse, and dry gently. Bond is applied and polymerized according to the steps in self-etch mode.
**Clearfil S^3^ Bond Universal—Kuraray, Okayama, Japan**	BisGMA, HEMA, ethanol, 10-MDP, hydrophilic aliphatic dimethacrylate, colloidal silica, dl-camphorquinone, silane coupling agent, accelerators, initiators, water **(pH: 2.7)**	**Self-etch** Apply the adhesive with rubbing for 10 s. Thin the adhesive on the surface with light air for 5 s. Cure with light sources at 1200 mW/cm^2^ for 10 s. **Total-etch** Phosphoric acid gel is applied for 30 s. Wash thoroughly and dry the surface by gently spraying it with air. Bond is applied and polymerized according to the steps in self-etch mode.
**FineEtch—Spident**	37% phosphoric acid gel (glycerol containing etchant) **(pH < 1)**	Before applying the adhesive, the acid is left intact for the time recommended by the manufacturer, and then the surface is washed and rinsed with water using an air–water spray.
**Filtek™ Ultimate Universal—3M ESPE; St Paul, MN, USA/A2**	BisGMA, BisEMA-6, UDMA, PEGDMA, TEGDMA, silane-treated ceramic filler, silica, zirconia, and catalysts (nanofilled composite)	Placed using the incremental technique (2 mm). Cured with light sources for 20 s.

**Table 2:** DIAGNOdent Pen group mean values (DIAGNOdent Pen reference values [23]: intact enamel: 0–6, enamel caries: 7–17, dentin caries: 18–99).

Groups	Initial Measurements MEAN ± STD	Measurements After Demineralization MEAN ± STD	Measurements After Remineralization MEAN ± STD
Group 1	2.2 ± 0.5	-	-
Group 2	2.1 ± 0.4	-	-
Group 3	2.1 ± 0.4	12.3 ± 1	-
Group 4	2.3 ± 0.5	12.8 ± 1.1	-
Group 5	2.1 ± 0.4	12.5 ± 0.9	3.8 ± 0.7
Group 6	2.4 ± 0.5	12.3 ± 1	3.9 ± 0.8
Group 7	2.1 ± 0.4	-	-
Group 8	2.4 ± 0.5	-	-
Group 9	2.1 ± 0.4	12.5 ± 0.8	-
Group 10	2.2 ± 0.5	12.4 ± 0.9	-
Group 11	2.3 ± 0.5	12.4 ± 0.9	3.9 ± 0.8
Group 12	2.4 ± 0.5	12.7 ± 1.1	3.7 ± 0.9

**Table 3:** Evaluation of shear bond strength according to different universal adhesives, enamel surfaces, and application modes. Note: Different letters in the lines indicate the differences between enamel surfaces. ^1^p: Evaluation result between enamel surfaces, ^2^p: evaluation result between application modes, ^3^p: evaluation result between universal adhesives (TE: total-etch; SE: self-etch).

Universal Adhesive	Application Mode	Intact Enamel	Demineralized Enamel	Remineralized Enamel	
		MEAN ± STD	MEAN ± STD	MEAN ± STD	^1^p
G-Premio Bond Universal	TE	8.79 ± 1.32 ^A^	3.74 ± 1.26 ^B^	7.53 ± 1.16 ^C^	0.001 *
	SE	7.26 ± 1.27 ^A^	3.44 ± 1.28 ^B^	6.96 ± 1.18 ^A^	0.001 *
	^2^p	0.009 *	0.565	0.242	
Clearfil S^3^ Bond Universal	TE	9.53 ± 1.42 ^A^	4.90 ± 1.08 ^B^	9.14 ± 1.20 ^A^	0.001 *
	SE	7.65 ± 1.39 ^A^	5.21 ± 1.28 ^B^	7.09 ± 1.35 ^A^	0.001 *
	^2^p	0.003 *	0.526	0.001 *	
G- Premio Bond Universal—Clearfil S^3^ Bond Universal	TE ^3^p	0.201	0.024 *	0.003 *	
	SE ^3^p	0.487	0.003 *	0.803	

Three-way ANOVA test * *p* < 0.05.

**Table 4:** Failure type analysis (I: intact enamel, D: demineralized enamel, R: remineralized enamel, G: G-Premio Bond Universal, C: Clearfil S^3^ Bond Universal, T: total-etch, S: self-etch).

Groups	Adhesive Failure	Cohesive Failure in Enamel	Cohesive Failure in Composite	Mixed Failure
Group 1 (I.G.T.)	66.6% (8/12)	-	8.33% (1/12)	25% (3/12)
Group 2 (I.G.S.)	50% (6/12)	-	-	50% (6/12)
Group 3 (D.G.T.)	33.33% (4/12)	25% (3/12)	-	41.66% (5/12)
Group 4 (D.G.S.)	25% (3/12)	33.33% (4/12)	-	41.66% (5/12)
Group 5 (R.G.T.)	50% (6/12)	-	8.33% (1/12)	41.66% (5/12)
Group 6 (R.G.S.)	41.66% (5/12)	8.33% (1/12)	-	50% (6/12)
Group 7 (I.C.T.)	75% (9/12)	-	8.33% (1/12)	&16.66 (2/12)
Group 8 (I.C.S.)	58.33% (7/12)	-	&16.66 (2/12)	25% (3/12)
Group 9 (D.C.T.)	33.33% (4/12)	33.33% (4/12)	-	33.33% (4/12)
Group 10 (D.C.S.)	33.33% (4/12)	25% (3/12)	-	41.66% (5/12)
Group 11 (R.C.T.)	50% (6/12)	8.33% (1/12)	&16.66 (2/12)	25% (3/12)
Group 12 (R.C.S.)	41.66% (5/12)	8.33% (1/12)	8.33% (1/12)	41.66% (5/12)

**Table 5:** Evaluation of Ca/P mineral atomic ratios according to universal adhesive, enamel surface, and application mode. Note: Different letters in the lines indicate the differences between enamel surfaces. ^1^p: Evaluation result between enamel surfaces, ^2^p: evaluation result between application modes, ^3^p: evaluation result between universal adhesives (TE: total-etch; SE: self-etch).

Universal Adheziv	Application Mode	Intact Enamel	Demineralized Enamel	Remineralized Enamel	
		MEAN ± STD	MEAN ± STD	MEAN ± STD	^1^p
G-Premio Bond Universal	TE	1.59 ±0.02 ^A^	1.39 ± 0.04 ^B^	1.54 ± 0.02 ^C^	0.001 *
	SE	1.66 ± 0.02 ^A^	1.52 ± 0.06 ^B^	1.57 ± 0.04 ^C^	0.001 *
	^2^p	0.001 *	0.001 *	0.062	
Clearfil S^3^ Bond Universal	TE	1.65 ± 0.05 ^A^	1.44 ± 0.05 ^B^	1.61 ± 0.04 ^A^	0.001 *
	SE	1.74 ± 0.03 ^A^	1.53 ± 0.03 ^B^	1.62 ± 0.03 ^C^	0.001 *
	^2^p	0.001 *	0.001 *	0.503	
G-Premio-Clearfil S^3^	TE ^3^p	0.006 *	0.037 *	0.001 *	
	SE ^3^p	0.001 *	0.466	0.002 *	

Three-way ANOVA test * *p* < 0.05.

**Table 6:** Evaluation of Ca/P mineral mass ratios according to universal adhesive, enamel surface, and application mode. Note: Different letters in the lines indicate the differences between enamel surfaces. ^1^p: Evaluation result between enamel surfaces, ^2^p: evaluation result between application modes, ^3^p: evaluation result between universal adhesives (TE: total-etch; SE: self-etch).

Universal Adheziv	Application Mode	Intact Enamel	Demineralized Enamel	Remineralized Enamel	
		MEAN ± STD	MEAN ± STD	MEAN ± STD	^1^p
G-Premio Bond Universal	TE	2.06 ± 0.03 ^A^	1.80 ± 0.05 ^B^	2.00 ± 0.02 ^C^	0.001 *
	SE	2.15 ± 0.03 ^A^	1.96 ± 0.07 ^B^	2.03 ± 0.05 ^C^	0.001 *
	^2^p	0.001 *	0.001 *	0.062	
Clearfil S^3^ Bond Universal	TE	2.13 ± 0.06 ^A^	1.86 ± 0.07 ^B^	2.09 ± 0.05 ^A^	0.001 *
	SE	2.25 ± 0.03 ^A^	1.98 ± 0.04 ^B^	2.10 ± 0.03 ^C^	0.001 *
	^2^p	0.001 *	0.001 *	0.503	
G-Premio-Clearfil S^3^	TE ^3^p	0.006 *	0.037 *	0.001 *	
	SE ^3^p	0.001 *	0.466	0.002 *	

Three-way ANOVA test * *p* < 0.05.

## Data Availability

The article itself contains the information needed to support its conclusions. Upon reasonable request, the corresponding author will make the dataset available.

## References

[1] Featherstone J.D. (2008). Dental caries: A dynamic disease process. Aust. Dent. J..

[2] Featherstone J.D.B. (1999). Prevention and reversal of dental caries: Role of low level fluoride. Community Dent. Oral. Epidemiol..

[3] Alafifi A., Yassen A.A., Hassanein O.E. (2019). Effectiveness of polyacrylic acid-bioactive glass air abrasion preconditioning with NovaMin remineralization on the microhardness of incipient enamel-like lesion. J. Conserv. Dent..

[4] Worawongvasu R. (2015). A Scanning Electron Microscopic Study of Enamel Surfaces of Incipient Caries. Ultrastruct. Pathol..

[5] Mobarak E.H., Ali N., Daifalla L.E. (2015). Microshear Bond Strength of Adhesives to Enamel Remineralized Using Casein Phosphopeptide Agents. Oper. Dent..

[6] Ghadirian H., Geramy A., Shallal W., Heidari S., Noshiri N., Keshvad M.A. (2020). The Effect of Remineralizing Agents With/Without CO2 Laser Irradiation on Structural and Mechanical Properties of Enamel and its Shear Bond Strength to Orthodontic Brackets. J. Lasers Med. Sci..

[7] Abdelmegid F.Y., Salama F.S., Abouobaid E.I., Halawany H.S., Alhadlaq M.K. (2019). Effect of Remineralizing Agents on Bond Strength of Resin-Composites to Primary Enamel. J. Clin. Pediatr. Dent..

[8] Borges A.B., Abu Hasna A., Matuda A.G.N., Lopes S.R., Mafetano A., Arantes A., Duarte A.F., Barcellos D.C., Torres C.R.G., Pucci C.R. (2019). Adhesive systems effect over bond strength of resin-infiltrated and de/remineralized enamel. F1000Res.

[9] Thimmaiah C., Shetty P., Shetty S.B., Natarajan S., Thomas N.A. (2019). Comparative analysis of the remineralization potential of CPP-ACP with Fluoride, Tri-Calcium Phosphate and Nano Hydroxyapatite using SEM/EDX—An in vitro study. J. Clin. Exp. Dent..

[10] Jayarajan J., Janardhanam P., Jayakumar P. (2011). Efficacy of CPP-ACP and CPP-ACPF on enamel remineralization—An in vitro study using scanning electron microscope and DIAGNOdent. Indian. J. Dent. Res..

[11] Iijima Y., Cai F., Shen P., Walker G., Reynolds C., Reynolds E.C. (2004). Acid resistance of enamel subsurface lesions remineralized by a sugar-free chewing gum containing casein phosphopeptide-amorphous calcium phosphate. Caries Res..

[12] Moule C.A., Angelis F., Kim G.H., Le S., Malipatil S., Foo M.S., Burrow M.F., Thomas D. (2007). Resin bonding using an all-etch or self-etch adhesive to enamel after carbamide peroxide and/or CPP-ACP treatment. Aust. Dent. J..

[13] Dos Reis B.C., Lacerda A.J.F.d., Canepele T.M.F., Borges A.B., Yui K.C.K., Torres C.R.G., Pucci C.R. (2016). Evaluation of bond strength of composite resin to enamel demineralized, exposed to remineralization and subjected to caries infiltration. Braz. Dent. Sci..

[14] Van Meerbeek B., Yoshihara K., Yoshida Y., Mine A., De Munck J., Van Landuyt K.L. (2011). State of the art of self-etch adhesives. Dent. Mater..

[15] Choi A.N., Lee J.H., Son S.A., Jung K.H., Kwon Y.H., Park J.K. (2017). Effect of Dentin Wetness on the Bond Strength of Universal Adhesives. Materials.

[16] Muñoz M.A., Luque-Martinez I., Malaquias P., Hass V., Reis A., Campanha N.H., Loguercio A.D. (2015). In vitro longevity of bonding properties of universal adhesives to dentin. Oper. Dent..

[17] Peumans M., De Munck J., Van Landuyt K.L., Poitevin A., Lambrechts P., Van Meerbeek B. (2010). Eight-year clinical evaluation of a 2-step self-etch adhesive with and without selective enamel etching. Dent. Mater..

[18] Frankenberger R., Lohbauer U., Roggendorf M.J., Naumann M., Taschner M. (2008). Selective enamel etching reconsidered: Better than etch-and-rinse and self-etch?. J. Adhes. Dent..

[19] Erickson R.L., Barkmeier W.W., Latta M.A. (2009). The role of etching in bonding to enamel: A comparison of self-etching and etch-and-rinse adhesive systems. Dent. Mater..

[20] Almarsomy D.H., Al-Khayat F.A., Al-Taee L.A. (2023). The preventive/therapeutic effect of CO(2) laser and MI Paste Plus^®^ on intact and demineralized enamel against Streptococcus mutans (In Vitro Study). Heliyon.

[21] Ortiz-Ruiz A.J., Munoz-Gomez I.J., Perez-Pardo A., German-Cecilia C., Martinez-Beneyto Y., Vicente A. (2018). Influence of fluoride varnish on shear bond strength of a universal adhesive on intact and demineralized enamel. Odontology.

[22] International Standard Adhesives—Designation of Main Failure Patterns. https://cdn.standards.iteh.ai/samples/80800/cc2769b3d44a498dbe129fa08906f944/ISO-10365-2022.pdf.

[23] Lussi A., Hellwig E. (2006). Performance of a new laser fluorescence device for the detection of occlusal caries in vitro. J. Dent..

[24] Perdigao J., Lopes M.M., Gomes G. (2008). In vitro bonding performance of self-etch adhesives: II--ultramorphological evaluation. Oper. Dent..

[25] Orilisi G., Vitiello F., Notarstefano V., Furlani M., Riberti N., Monterubbianesi R., Bellezze T., Campus G., Carrouel F., Orsini G. (2023). Multidisciplinary evaluation of the remineralization potential of three fluoride-based toothpastes on natural white spot lesions. Clin. Oral. Investig..

[26] Cardenas A.F.M., Armas-Veja A., Rodriguez Villarreal J.P., Siqueira F.S.F., Muniz L.P., Campos V.S., Reis A., Loguercio A.D. (2019). Influence of the mode of application of universal adhesive systems on adhesive properties to fluorotic enamel. Braz. Oral. Res..

[27] Wang C., Fang Y., Zhang L., Su Z., Xu J., Fu B. (2021). Enamel microstructural features of bovine and human incisors: A comparative study. Ann. Anat..

[28] Attin R., Stawarczyk B., Kecik D., Knosel M., Wiechmann D., Attin T. (2012). Shear bond strength of brackets to demineralize enamel after different pretreatment methods. Angle Orthod..

[29] Lopes M.B., Sinhoreti M.A., Correr Sobrinho L., Consani S. (2003). Comparative study of the dental substrate used in shear bond strength tests. Pesqui. Odontol. Bras..

[30] Reis A.F., Giannini M., Kavaguchi A., Soares C.J., Line S.R. (2004). Comparison of microtensile bond strength to enamel and dentin of human, bovine, and porcine teeth. J. Adhes. Dent..

[31] Fowler C.S., Swartz M.L., Moore B.K., Rhodes B.F. (1992). Influence of selected variables on adhesion testing. Dent. Mater..

[32] Wang C., Li Y., Wang X., Zhang L., Tiantang, Fu B. (2012). The enamel microstructures of bovine mandibular incisors. Anat. Rec..

[33] Davidson C.L., Boom G., Arends J. (1973). Calcium distribution in human and bovine surface enamel. Caries Res..

[34] Feagin F., Koulourides T., Pigman W. (1969). The characterization of enamel surface demineralization, remineralization, and associated hardness changes in human and bovine material. Arch. Oral. Biol..

[35] Goncalves F.M.C., Delbem A.C.B., Gomes L.F., Emerenciano N.G., Dos Passos Silva M., Cannon M.L., Danelon M. (2021). Combined effect of casein phosphopeptide-amorphous calcium phosphate and sodium trimetaphosphate on the prevention of enamel demineralization and dental caries: An in vitro study. Clin. Oral. Investig..

[36] Escalante-Otarola W.G., Castro-Nunez G.M., Leandrim T.P., Alencar C.M., de Albuquerque Jasse F.F., Kuga M.C. (2021). Effects of Remineralizing Agents Based on Calcium Phosphate, Sodium Phosphate, or Sodium Fluoride on Eroded Cervical Dentin. Oper. Dent..

[37] Samuel S.R., Dorai S., Khatri S.G., Patil S.T. (2016). Effect of ozone to remineralize initial enamel caries: In situ study. Clin. Oral. Investig..

[38] Vinod D., Gopalakrishnan A., Subramani S.M., Balachandran M., Manoharan V., Joy A. (2020). A comparative evaluation of remineralizing potential of three commercially available remineralizing agents: An in vitro study. Int. J. Clin. Pediatr. Dent..

[39] Uysal T., Baysal A., Uysal B., Aydinbelge M., Al-Qunaian T. (2011). Do fluoride and casein phosphopeptide-amorphous calcium phosphate affect shear bond strength of orthodontic brackets bonded to a demineralized enamel surface?. Angle Orthod..

[40] Rashid M.F., Karobari M.I., Halim M.S., Noorani T.Y. (2022). Effectiveness of Visual-Tactile Examination and DIAGNOdent Pen in Detecting Early Enamel Caries and Its Remineralisation: An In Vitro Study. Biomed. Res. Int..

[41] Tulumbaci F., Oba A.A. (2019). Efficacy of different remineralization agents on treating incipient enamel lesions of primary and permanent teeth. J. Conserv. Dent..

[42] Lussi A., Imwinkelried S., Pitts N., Longbottom C., Reich E. (1999). Performance and reproducibility of a laser fluorescence system for detection of occlusal caries in vitro. Caries Res..

[43] Lussi A., Megert B., Longbottom C., Reich E., Francescut P. (2001). Clinical performance of a laser fluorescence device for detection of occlusal caries lesions. Eur. J. Oral. Sci..

[44] Josey A.L., Meyers I.A., Romaniuk K., Symons A.L. (1996). The effect of a vital bleaching technique on enamel surface morphology and the bonding of composite resin to enamel. J. Oral. Rehabil..

[45] Van Meerbeek B., Van Landuyt K., De Munck J., Hashimoto M., Peumans M., Lambrechts P., Yoshida Y., Inoue S., Suzuki K. (2005). Technique-sensitivity of contemporary adhesives. Dent. Mater. J..

[46] Van Landuyt K.L., Snauwaert J., De Munck J., Coutinho E., Poitevin A., Yoshida Y., Suzuki K., Lambrechts P., Van Meerbeek B. (2007). Origin of interfacial droplets with one-step adhesives. J. Dent. Res..

[47] Monticelli F., Osorio R., Pisani-Proença J., Toledano M. (2007). Resistance to degradation of resin-dentin bonds using a one-step HEMA-free adhesive. J. Dent..

[48] Torkabadi S., Nakajima M., Ikeda M., Foxton R.M., Tagami J. (2008). Bonding durability of HEMA-free and HEMA-containing one-step adhesives to dentine surrounded by bonded enamel. J. Dent..

[49] Takahashi M., Nakajima M., Hosaka K., Ikeda M., Foxton R.M., Tagami J. (2011). Long-term evaluation of water sorption and ultimate tensile strength of HEMA-containing/-free one-step self-etch adhesives. J. Dent..

[50] Sugimura R., Tsujimoto A., Hosoya Y., Fischer N.G., Barkmeier W.W., Takamizawa T., Latta M.A., Miyazaki M. (2019). Surface moisture influence on etch-and-rinse universal adhesive bonding. Am. J. Dent..

[51] Baysal A., Uysal T. (2012). Do enamel microabrasion and casein phosphopeptide-amorphous calcium phosphate affect shear bond strength of orthodontic brackets bonded to a demineralized enamel surface?. Angle Orthod..

[52] Moura S.K., Reis A., Pelizzaro A., Dal-Bianco K., Loguercio A.D., Arana-Chavez V.E., Grande R.H. (2009). Bond strength and morphology of enamel using self-etching adhesive systems with different acidities. J. Appl. Oral. Sci..

[53] Kamath P., Nayak R., Kamath S.U., Pai D. (2017). A comparative evaluation of the remineralization potential of three commercially available remineralizing agents on white spot lesions in primary teeth: An in vitro study. J. Indian Soc. Pedod. Prev. Dent..

